# Supplementary material for: Analysis of PPARγ Signaling Activity in Psoriasis
Source: Int J Mol Sci. 2021 Aug 10;22(16):8603. doi: 10.3390/ijms22168603 (PMC8395241; doi:10.3390/ijms22168603)
Supplement: Supplementary file 1 [file ijms-22-08603-s001.zip › Supplemental materials_Analysis of PPARg signaling activity in psoriasis/Pathway models/Models images and html files/Model of PPARG signaling in psoriasis/8941.html]

PLCG1


# Protein PLCG1

|  |  |
| --- | --- |
| URN | urn:agi-llid:5335 |
| Total Entities | 0 |
| Connectivity | 2314 |
| Name | PLCG1 |
| Description | phospholipase C, gamma 1 |
| Notes | The protein encoded by this gene catalyzes the formation of inositol 1,4,5-trisphosphate and diacylglycerol from phosphatidylinositol 4,5-bisphosphate. This reaction uses calcium as a cofactor and plays an important role in the intracellular transduction of receptor-mediated tyrosine kinase activators. For example, when activated by SRC, the encoded protein causes the Ras guanine nucleotide exchange factor RasGRP1 to translocate to the Golgi, where it activates Ras. Also, this protein has been shown to be a major substrate for heparin-binding growth factor 1 (acidic fibroblast growth factor)-activated tyrosine kinase. Two transcript variants encoding different isoforms have been found for this gene. [provided by RefSeq, Jul 2008] |
| Primary Cell Localization | Cytoplasm |

---

|  |  |
| --- | --- |
| Pathway | Skin Aging |
|  | I-cell: CCK-8 Secretion and Regulation of Eating Behavior |
|  | Dopamine Receptors Signaling |
|  | Thyroid Hormones Common non-Genomic Effects in Hyperthyroidism |
|  | Dopamine Synthesis and Release |
|  | Thrombopoietin Receptors Signaling in Platelet Maturation |
|  | T-Cell Independent B-Cell Activation |
|  | V(D)J Recombination Activation |
|  | Natural Killer Cell Activation through ITAM-Containing Receptors |
|  | Natural Killer Cell Inhibitory Receptor Signaling |
|  | Natural Killer Cell Activation through ITSM-Containing Receptors |
|  | T-Cell Maturation (Hypothesis) |
|  | Natural Killer Cell Activation |
|  | Peripheral T-Cell Tolerance |
|  | T-Cell Central Tolerance |
|  | CD8+ T-Cell Activation |
|  | Th1-Cell Differentiation |
|  | Treg-Cell Differentiation |
|  | Th2-Cell Differentiation |
|  | T-Cell Receptor Signaling |
|  | Th17-Cell Differentiation |
|  | Platelet Activation via Adhesion Molecules |
|  | Proplatelet Maturation |
|  | Eat me Signal: Apoptotic Cell Induces Phagocytosis |
|  | Basophil Activation |
|  | Eosinophil Activation |
|  | Mast-Cell Activation via IgE Signaling |
|  | Mast-Cells De Novo Synthesized Mediators via IgE Independent Signaling |
|  | CD16/CD14 Proinflammatory Monocyte Activation |
|  | Macrophage M2-Related Phagocytosis |
|  | anti-Inflammatory Function of Macrophage M2 Lineage |
|  | Neutrophil Activation via FCGR3B |
|  | Neutrophil Degranulation via FPR1 Signaling |
|  | CR3 Mediated Phagocytosis in Neutrophils and Macrophages |
|  | Neutrophil Activation via Adherence on Endothelial Cells |
|  | Vascular Endothelial Cell Activation by NO |
|  | Vascular Smooth Muscle Cell/Pericyte Migration and Proliferation |
|  | Vascular Endothelial Cell Activation by Growth Factors |
|  | Vascular Endothelial Cell Permeability Activation |
|  | Potassium Flux Regulation |
|  | GFs/TNF -> Ion Channels |
|  | Nociception Expression Targets Signaling |
|  | Ca2+ Flux Regulation |
|  | CRH -> Synthesis of Corticosteroids |
|  | DRD2 Expression Targets |
|  | DRD2/4 -> Membrane Transport |
|  | NTRK1/2/3 -> Acetylcholine Production |
|  | EPHB -> NMDA Receptor Activation |
|  | non-Genomic Rapid Actions of Vitamin D in Vitamin D Biology |
|  | G0/G1 Phase Transition |
|  | Th1-Cells Activation and Proliferation in Atherosclerosis |
|  | Dilated Cardiomyopathy |
|  | Smooth Muscle Cell Dysfunction in Pulmonary Hypertension |
|  | Lymphocyte Mediated Myocardial Injury in Myocarditis |
|  | Neurons Necrosis Caused by Energy Deficiency |
|  | Contraction Due Vasospasm |
|  | Sebocyte Proliferation in Acne Vulgaris |
|  | Atopic Dermatitis Onset |
|  | Cell Cycle Dysregulation in Melanoma |
|  | WNT Signaling in Melanoma |
|  | Melanoma |
|  | Apoptosis Block in Melanoma Cell |
|  | MITF as a Regulator of Melanoma Cell Development |
|  | Th0-Cell Aberrant Activation in Systemic Lupus Erythematosus |
|  | Th17-Cell Function in Systemic Lupus Erythematosus |
|  | Th1-Cell Function in Systemic Lupus Erythematosus |
|  | Th2-Cell Function in Systemic Lupus Erythematosus |
|  | Acute Cytotoxic CD8+T-Cell Response against Melanocytes |
|  | Vitiligo |
|  | T-Cell Cytotoxic Response against Melanocytes in Vitiligo |
|  | Apoptosis of Melanocyte in Vitiligo |
|  | CD8+ T-Cell Response to Self-Determinants in Diabetes |
|  | Treg-Cell Function in Diabetes Mellitus Type 1 |
|  | Natural Killer T-Cell Roles in Diabetes Mellitus Type 1 |
|  | non-Suppressive Treg-Cell in Diabetes Mellitus Type 1 |
|  | TLR2 Signaling in Treg-Cell in Type 1 Diabetes (Animal Model) |
|  | Natural Killer Cell in Diabetes Mellitus Type 1 |
|  | Peripheral Tolerance to Autoantigens Recession in Diabetes Mellitus Type 1 |
|  | Treg-Cell Activation in Diabetes Mellitus |
|  | Proliferative Diabetic Retinopathy |
|  | Immune Sustem Activation in Hashimoto's Thyroiditis |
|  | Hashimoto's Thyroiditis |
|  | RET1 Mutation in Hyperparathyroidism |
|  | Cardiovascular Effects in Hyperthyroidism |
|  | Immune System Activation in Hyperthyroidism |
|  | Hypothyroidism, Secondary (Central) |
|  | Prolactinoma Overiew |
|  | CD4+T-Cell Response in Celiac Disease |
|  | CD8+ T-Cell Response in Celiac Disease |
|  | B-Cell Activation in Crohn's Disease |
|  | OipA Signaling in Helicobacter Pylori Infection |
|  | CagA Phosphorylation Independent Signaling |
|  | EDN, NRG, NRTN, and GDNF/RET Signaling in Hirschsprung Disease |
|  | Th2-Cell Function in Ulcerative Colitis |
|  | T-Cell Cytotoxic Response in Ulcerative Colitis |
|  | Hodgkin Lymphoma |
|  | T-Cell Acute Lymphoblastic Leukemia |
|  | Goblet-Cell Exocytosis in Asthma |
|  | Neutrophil Degranulation via FPR1/IL8 |
|  | Basophil Activation in Asthma |
|  | Mast-Cell Activation in Asthma |
|  | Antigen-Presenting Cell Role in Asthma |
|  | Th17-Cell Differentiation in Asthma |
|  | Th2-Cell Response in Asthma |
|  | CFTR Facilitates Oxidative Stress in Airway Epithelium in CF |
|  | Osteoclast Activation in Gout |
|  | Th17-Cell and Th1 Immune Responsein Psoriatic Arthritis |
|  | PTPN22 Role in Rheumatoid Arthritis |
|  | Osteoclast Activation in Rheumatoid Arthritis |
|  | Th2-Cells Function in Systemic Scleroderma |
|  | Dentin Formation Disruption |
|  | CD8+ T-Cell Action Impairment in HIV Type 1 Infection |
|  | CD4+ T-Cell Function Decline in HIV |
|  | CD4+ T-Cell Death in HIV |
|  | Glutamate Toxiicity Effects on Striatum Medium Spiny Neurons in Hantington's Disease |
|  | BDNF Upregulation Triggered by Seizures |
|  | Glioblastoma, Primary |
|  | Proteins Involved in Glioblastoma |
|  | Glioblastoma, Secondary |
|  | Astrocytoma |
|  | Dopamine Synthesis and Release in Migraine |
|  | Postsynaptic Neuron Activation |
|  | Brain Cell Necrosis in Vascular Dementia |
|  | Angiogenesis in Endometriosis |
|  | HPV E5 Regulates EDNRA and EGFR Signaling Pathway in Keratinocytes |
|  | Oxidative Stress, All-Trans-Retinal and Lipofuscin Toxicity in AMD |
|  | Ca2+ Toxicity in Lens Cells |
|  | VEGFA in Neovascular Glaucoma |
|  | Cytokines Trigger Otitis Media |
|  | VEGFA Low Expression Level in Chronic Bronchitis |
|  | VEGFA High Expression Level in Pulmonary Emphysema |
|  | Penile Detumescense Augmentation Mechanisms |
|  | CD38/CD3 -> JUN/FOS/NF-kB Signaling in T-cell Proliferation |
|  | CD157 -> cADPR/Calcium Signaling |
|  | ANGPT1 -> CREB/CREBBP Expression Targets |
|  | ANGPT2 -> AP-1/CREBBP/MYC Expression Targets |
|  | AREG -> CREB/CREBBP Expression Targets |
|  | AREG -> NCOR2 Expression Targets |
|  | AREG -> SMAD1 Expression Target |
|  | AREG -> HIF1A Expression Targets |
|  | AREG -> AP-1 Expression Targets |
|  | Dopamine/Gi Expression Targets |
|  | BTC -> AP-1/ATF/CREB Expression Targets |
|  | BTC -> EP300/ETS/ETV/SP1 Expression Targets |
|  | SELE -> ELK-SRF Signaling |
|  | CD2 Expression Targets |
|  | CD247 Expression Targets |
|  | FCGR3A Expression Targets |
|  | CD22 -> NF-kB Expression Targets |
|  | CD8 -> AP-1 Expression Targets |
|  | CD72 -> CREB/CREBBP Expression Targets |
|  | CD72 -> NF-kB Expression Targets |
|  | CD72 -> STAT Expression Target |
|  | CD8 -> ATF/CREB/CREBBP Expression Targets |
|  | CD8 -> NF-kB Expression Targets |
|  | CD8 -> STAT Expression Targets |
|  | CD80 -> AP-1 Expression Targets |
|  | CD80 -> STAT Expression Targets |
|  | CD81 Expression Targets |
|  | CD80 -> NF-kB Expression Targets |
|  | CD80 -> NFATC Expression Targets |
|  | CD86 -> NFATC Expression Targets |
|  | CD86 -> STAT Expression Targets |
|  | CD86-> NFATC Expression Targets |
|  | CD86 -> NF-kB Expression Targets |
|  | Doxorubicin Induced Cardiotoxicity |
|  | EGF -> HIF1A Expression Targets |
|  | EGF -> AP-1/ATF Expression Targets |
|  | EGF -> CREB/CREBBP/ELK/SRF/MYC Expression Targets |
|  | EGF -> TP53 Expression Targets |
|  | EGF -> NCOR2 Expression Target |
|  | EGFR -> NCOR2 Signaling |
|  | EGFR/ERBB2 -> TP53 Signaling |
|  | ERBB2/3 -> EP300/ETS/ETV/SP1 Signaling |
|  | EGFR -> SMAD1 Signaling |
|  | EGFR/ERBB2 -> HIF1A Signaling |
|  | EGFR -> AP-1/ATF2 Signaling |
|  | EGFR -> AP-1/CREB/ELK/SRF/MYC Signaling |
|  | EREG -> CREB Expression Target |
|  | EREG -> EP300/SP1 Expression Targets |
|  | EREG -> HIF1A Expression Target |
|  | EREG -> AP-1/ATF Expression Targets |
|  | Erythropoietin -> AP-1/MYC/CREB Expression Targets |
|  | FGF9 -> AP-1/CREB/MYC Expression Targets |
|  | FGF10 -> AP-1/CREB/CREBBP/MYC Expression Targets |
|  | FGF1 -> AP-1/CREB/ELK/SRF/MYC Expression Targets |
|  | FGF1 -> NCOR2 Expression Target |
|  | FGF7 -> RUNX Expression Targets |
|  | FGF8 -> AP-1/CREB/MYC Expression Targets |
|  | FGF1 -> RUNX Expression Targets |
|  | FGF18 -> AP-1/CREB Expression Targets |
|  | FGF23 -> NCOR2 Expression Targets |
|  | FGF8 -> RUNX Expression Targets |
|  | FGF9 -> RUNX Expression Targets |
|  | FGF2 -> NCOR2 Expression Targets |
|  | FGF4 -> AP-1/MYC Expression Targets |
|  | FGF7 -> AP-1/CREB/CREBBP/MYC Expression Targets |
|  | FGF2 -> RUNX Expression Targets |
|  | FGF2 -> AP-1/CREB/CREBBP/ELK/SRF/MYC Expression Targets |
|  | FGFR -> RUNX2 Signaling |
|  | FGFR -> AP-1/CREB/CREBBP/ELK/SRF/MYC Signaling |
|  | FPR1 -> Cytoskeleton Signaling |
|  | HBEGF -> AP-1/ATF Expression Targets |
|  | HBEGF -> TP53 Expression Targets |
|  | HBEGF -> EP300/ETS/ETV/SP1 Expression Targets |
|  | HBEGF -> HIF1A Expression Targets |
|  | HBEGF -> CREB/MYC Expression Targets |
|  | HGF -> AP-1/CREB/ELK/SRF/MYC Expression Targets |
|  | HGFR -> AP-1/CREB/MYC Signaling |
|  | TAM Receptor Signaling Activation in Cancer |
|  | Receptors and Adaptor Proteins Activated in Cancer |
|  | Proteins with Altered Expression in Cancer-Associated Sustaining of Proliferative Signaling |
|  | Genes with Mutations in Cancer-Associated Sustaining of Proliferative Signaling |
|  | G0/G1 Cell Cycle Phase Transition Activation in Cancer |
|  | VEGF Independent Angiogenesis in Cancer |
|  | CDH2 Activation Promotes Cancer Cell Migration and Survival |
|  | CFL1 in Cancer Cell Motility |
|  | Treg Cells Promote Immunosuppression in Cancer Immune Escape |
|  | Effector T-cell Inactivation in Cancer Immune Escape |
|  | CD2 -> NFATC1 Signaling |
|  | FcIgER -> NFATC1 Signaling |
|  | FcIgER -> ELK-SRF Signaling |
|  | ICAM1 -> AP-1/CREB/ELK/SRF Signaling |
|  | FibronectinR -> AP-1/ELK/SRF/SREBF Signaling |
|  | CTGF -> AP-1/CREB/MYC Expression Targets |
|  | CTGF -> NCOR2 Expression Target |
|  | ICAM1 Expression Targets |
|  | IL16 -> AP-1 Expression Targets |
|  | IL16-> ATF/CREB/CREBBP Expression Target |
|  | IL16 -> NF-kB Expression Targets |
|  | UrokinaseR -> ELK/SRF Signaling |
|  | PTPRC -> BCL6 Expression Targets |
|  | LPA Expression Targets |
|  | NRG1 -> EP300/ETS/ETV/SP1 Expression Targets |
|  | NRG1 -> CREB/CREBBP/ELK/SRF/MYC Expression Targets |
|  | NRG1 -> TP53 Expression Target |
|  | NRG1 -> AP-1/ATF Expression Targets |
|  | NRG1 -> HIF1A Expression Target |
|  | NGF -> SMAD3/NF-kB Expression Targets |
|  | NGF -> AP-1/TP53/MYC Expression Targets |
|  | NGF -> FOXO/MYCN/ELK-SRF Expression Targets |
|  | BDNF Expression Targets |
|  | NTF3 Expression Targets |
|  | NTF4 Expression Targets |
|  | NGF -> CREB/CEBPB/MEF2A Expression Targets |
|  | PDGFB -> AP-1/CREB/MYC Expression Target |
|  | PDGFC -> CREB Expression Target |
|  | PDGFD -> AP-1 Expression Targets |
|  | PDGF -> AP-1/CREB/CREBBP/MYC Expression Targets |
|  | KIT -> MITF Signaling |
|  | PDGFR -> AP-1/MYC Signaling |
|  | PDCD1 -> ATF/CREB/CREBBP Expression Targets |
|  | PDCD1 -> NF-kB Expression Targets |
|  | PDCD1 -> NFATC Expression Targets |
|  | PDCD1 -> AP-1 Expression Targets |
|  | PTPRC -> BCL6 Signaling |
|  | AngiopoietinR -> AP-1 Signaling |
|  | GDNF -> HSF1 Signaling |
|  | NTRK -> AP-1/CREB/ELK/SRF/MYC/SMAD3/TP53 Signaling |
|  | GAS6 -> AP-1/CREB Expression Targets |
|  | FIGF -> AP-1 Expression Target |
|  | FIGF -> NCOR2 Expression Target |
|  | KITLG -> AP-1/CREB/CREBBP/MYC Expression Targets |
|  | GDNF -> HSF1 Expression Targets |
|  | CSF1 -> AP-1/CREB/CREBBP/MYC Expression Targets |
|  | PGF -> AP-1/CREB/CREBBP/MYC Expression Targets |
|  | FLT3LG -> AP-1/CREB/CREBBP Expression Targets |
|  | AXL Receptor Tyrosine Kinase Signaling |
|  | VEGF Signaling |
|  | EGFR Signaling |
|  | TCR -> CREB/CREBBP/ATF Expression Targets |
|  | TCR -> NF-kB Expression Targets |
|  | TCR -> NFAT Expression Targets |
|  | TCR -> AP-1 Expression Targets |
|  | T-Cell Receptor -> AP-1 Signaling |
|  | T-Cell Receptor -> NF-kB Signaling |
|  | T-Cell Receptor -> NFATC Signaling |
|  | T-Cell Receptor -> CREBBP Signaling |
|  | TGFA -> TP53 Expression Targets |
|  | TGFA -> HIF1A Expression Targets |
|  | TGFA/AP-1/ATF Expression Targets |
|  | TGFA -> CREB/CREBBP/ELK-SRF/MYC Expression Targets |
|  | Thrombopoietin -> AP-1/CREB/CREBBP/MYC Expression Targets |
|  | Ca2+ Cytosolic Overload |
|  | PLAU -> ELK-SRF/AP-1 Expression Targets |
|  | VEGFA -> ATF/CREB/ELK-SRF Expression Targets |
|  | VEGFA -> NFATC Expression Targets |
|  | VEGFC -> ATF Expression Target |
|  | VEGFA -> AP-1/CREBBP/MYC Expression Targets |
|  | VEGFA -> NCOR2 Expression Target |
|  | VEGFR -> NFATC Signaling |
|  | VEGFR -> AP-1/CREB/MYC Signaling |
|  | VEGFR -> ATF/CREB/ELK-SRF Signaling |
|  | sialic |
|  | cypa |
|  | Model of PPARG signaling in psoriasis |
|  | New Pathway |
|  | 2\_Hyperseborrhea in acne vulgaris |
|  | 1\_The onset of atopic dermatitis |
|  | 3\_Enhanced angiogenesis in endometriosis |
|  | 2\_Oxidative stress, all-trans-retinal, and lipofuscin toxicity in agerelated macular degeneration |
|  | 2\_Pathogens stimulate mucins expression in the middle ear |
|  | 3\_1\_Mucus accumulation in asthma |
|  | 4\_Mucin hyperproduction in goblet and mucous cells in COPD |
|  | 1\_CD4+ T cell and epithelial cell response in celiac disease |
|  | 2\_CD8+ T-cell response and mucosal damage in celiac disease |
|  | 3\_Islet beta-cell destruction in T1D |
|  | 2\_1\_General pathways of thyroid hormone signaling |
|  | drug-target pairs binding |

---

|  |  |
| --- | --- |
| Source | curated |
|  | GO component |
|  | Biopax |

---

|  |  |
| --- | --- |
| MedScan ID | 5335 |

---

|  |  |
| --- | --- |
| LocusLink ID | 5335 |
|  | 18803 |
|  | 25738 |
|  | 381402 |
|  | 99130 |
|  | 12547 |
|  | 281987 |
|  | 373867 |
|  | 336067 |
|  | 336221 |

---

|  |  |
| --- | --- |
| Alias | phospholipase C-II |
|  | PIG1\_HUMAN |
|  | PLC-II |
|  | 1-phosphatidylinositol-4,5-bisphosphate phosphodiesterase gamma I |
|  | PLCgamma1 |
|  | PLC-gamma I |
|  | Phospholipase C-gamma |
|  | AI894140 |
|  | Phospholipase C-gamma I |
|  | Plcg I |
|  | NCKAP3 |
|  | phospholipase C-148 |
|  | OTTMUSP00000017125 |
|  | PPLCA |
|  | PLC148 |
|  | RP23-393F23.4 |
|  | PLC1 |
|  | LOC381402 |
|  | phosphoinositide phospholipase C-gamma-1 |
|  | OTTHUMP00000178982 |
|  | 1-phosphatidylinositol-4,5-bisphosphate phosphodiesterase gamma 1 |
|  | PLC I |
|  | OTTHUMP00000031787 |
|  | PLC-gamma |
|  | PLCG1 |
|  | OTTMUSP00000001135 |
|  | PIG1\_RAT |
|  | 1-phosphatidyl-D-myo-inositol-4,5-bisphosphate |
|  | PLC-148 |
|  | PLC-gamma-1 |
|  | inositoltrisphosphohydrolase |
|  | monophosphatidylinositol phosphodiesterase |
|  | phosphatidylinositol phospholipase C |
|  | phosphoinositidase C |
|  | phosphoinositide phospholipase C |
|  | phospholipase C, gamma 1 (formerly subtype 148) |
|  | triphosphoinositide phosphodiesterase |
|  | 1-phosphatidylinositol 4,5-bisphosphate phosphodiesterase gamma-1 |
|  | Cded |
|  | Plc-1 |
|  | Plcg-1 |
|  | Plc-gamma1 |
|  | cell differentiation and embryonic development |
|  | 1-phosphatidylinositol-4,5-bisphosphate phosphodiesterase gamma-1 |
|  | phospholipase C-gamma-1 |
|  | phospholipase C gamma 1 |
|  | RP3-511B24.2 |
|  | PIG I |
|  | PIG1 |
|  | 1-phosphatidyl-D-myo-inositol-4,5-bisphosphate phosphodiesterase |
|  | PLC-148s |
|  | phospholipase C, gamma 1 |
|  | cell differentiating and embryonic development |

---

|  |  |
| --- | --- |
| EC Number | 3.1.4.11 |
|  | 3.1.4.3 |

---

|  |  |
| --- | --- |
| GO ID | 0005509 |
|  | 0035254 |
|  | 0005168 |
|  | 0004435 |
|  | 0004629 |
|  | 0019901 |
|  | 0030971 |
|  | 0038095 |
|  | 0038096 |
|  | 0050852 |
|  | 0007202 |
|  | 0007411 |
|  | 0019722 |
|  | 0016477 |
|  | 0071364 |
|  | 0007173 |
|  | 0001701 |
|  | 0043647 |
|  | 0032959 |
|  | 0050900 |
|  | 0050804 |
|  | 0048015 |
|  | 0009395 |
|  | 0045766 |
|  | 0043536 |
|  | 2000353 |
|  | 0010634 |
|  | 0010863 |
|  | 0051281 |
|  | 1905564 |
|  | 0051209 |
|  | 0007165 |
|  | 0016032 |
|  | 0008180 |
|  | 0098685 |
|  | 0042995 |
|  | 0005911 |
|  | 0005737 |
|  | 0005829 |
|  | 0098978 |
|  | 0030027 |
|  | 0005886 |
|  | 0001726 |
|  | 0016787 |
|  | 0005158 |
|  | 0046872 |
|  | 0051219 |
|  | 0008081 |
|  | 0006816 |
|  | 0035556 |
|  | 0016042 |
|  | 0006629 |
|  | 0009306 |
|  | 1901339 |
|  | 0030136 |
|  | 0043005 |
|  | 0009629 |
|  | 0042542 |
|  | 0043278 |
|  | 0010243 |
|  | 0005057 |
|  | 0000186 |
|  | 0004871 |
|  | 0007596 |
|  | 0019221 |
|  | 0008543 |
|  | 0045087 |
|  | 0048011 |
|  | 0044281 |
|  | 0048010 |
|  | 0019048 |
|  | 0005543 |
|  | 0005622 |
|  | 0044419 |
|  | 0032236 |
|  | 0023034 |
|  | 0016192 |
|  | 0007242 |
|  | 0005575 |
|  | 0005515 |
|  | 0008372 |
|  | 0001570 |
|  | 0008016 |
|  | 0031101 |

---

|  |  |
| --- | --- |
| KEGG ID | hsa:5335 |
|  | mmu:18803 |
|  | rno:25738 |

---

|  |  |
| --- | --- |
| MedLine Reference | 10913276 |
|  | 11724770 |
|  | 11823862 |
|  | 11886851 |
|  | 11989979 |
|  | 12009430 |
|  | 12437926 |
|  | 12507498 |
|  | 12623065 |
|  | 12646582 |
|  | 12850505 |
|  | 12930795 |
|  | 14523024 |
|  | 14568990 |
|  | 14570902 |
|  | 15060019 |
|  | 15161916 |
|  | 15169852 |
|  | 15252117 |
|  | 15258148 |
|  | 15536495 |
|  | 15579910 |
|  | 15607032 |
|  | 15607753 |
|  | 15744307 |
|  | 16819285 |
|  | 16841466 |
|  | 16989733 |
|  | 2840660 |
|  | 9892736 |
|  | 10191086 |
|  | 10349636 |
|  | 10373493 |
|  | 11042159 |
|  | 11076861 |
|  | 11217851 |
|  | 11438667 |
|  | 11544199 |
|  | 11562428 |
|  | 11744703 |
|  | 11748152 |
|  | 11916959 |
|  | 11931670 |
|  | 11934646 |
|  | 11973363 |
|  | 12061819 |
|  | 12065840 |
|  | 12149210 |
|  | 12374739 |
|  | 12466851 |
|  | 12477932 |
|  | 12761849 |
|  | 12909648 |
|  | 13129935 |
|  | 1358284 |
|  | 1409582 |
|  | 14612947 |
|  | 14687726 |
|  | 15006698 |
|  | 15062102 |
|  | 15121188 |
|  | 15196926 |
|  | 15214048 |
|  | 15329338 |
|  | 15367584 |
|  | 15372077 |
|  | 15563458 |
|  | 15595842 |
|  | 15657076 |
|  | 15782199 |
|  | 15817477 |
|  | 15944397 |
|  | 15953601 |
|  | 16141072 |
|  | 16141073 |
|  | 16319178 |
|  | 16537572 |
|  | 16571757 |
|  | 16670015 |
|  | 17015722 |
|  | 17114464 |
|  | 17128263 |
|  | 2033248 |
|  | 7744830 |
|  | 7774933 |
|  | 7781998 |
|  | 8672127 |
|  | 8687404 |
|  | 8739298 |
|  | 8889548 |
|  | 9096335 |
|  | 9472710 |
|  | 9529375 |
|  | 9662423 |
|  | 9685408 |
|  | 9691180 |
|  | 10350061 |
|  | 10430888 |
|  | 10455176 |
|  | 10467411 |
|  | 10473113 |
|  | 10501179 |
|  | 10586033 |
|  | 10652211 |
|  | 10692392 |
|  | 10770799 |
|  | 10783152 |
|  | 10823839 |
|  | 10843712 |
|  | 10873601 |
|  | 10899172 |
|  | 10926122 |
|  | 10940929 |
|  | 10980614 |
|  | 11043766 |
|  | 11071635 |
|  | 11094073 |
|  | 11259588 |
|  | 11312102 |
|  | 11368773 |
|  | 11390470 |
|  | 11390650 |
|  | 11409699 |
|  | 11779129 |
|  | 11941371 |
|  | 11956228 |
|  | 12071848 |
|  | 12084069 |
|  | 12085993 |
|  | 12163161 |
|  | 12419535 |
|  | 12454018 |
|  | 12482669 |
|  | 12496421 |
|  | 12514734 |
|  | 12598525 |
|  | 12601080 |
|  | 12771140 |
|  | 12788694 |
|  | 12845332 |
|  | 12890670 |
|  | 12937273 |
|  | 12960351 |
|  | 1383690 |
|  | 14647465 |
|  | 14702343 |
|  | 14722116 |
|  | 14989173 |
|  | 1500851 |
|  | 15123640 |
|  | 15144186 |
|  | 15153499 |
|  | 15184383 |
|  | 15556869 |
|  | 15623534 |
|  | 15654973 |
|  | 15766521 |
|  | 15872086 |
|  | 15951569 |
|  | 15972651 |
|  | 15996687 |
|  | 16341594 |
|  | 1639386 |
|  | 1676673 |
|  | 1683701 |
|  | 1689310 |
|  | 16921170 |
|  | 17023658 |
|  | 17111345 |
|  | 17130290 |
|  | 1715690 |
|  | 2167438 |
|  | 3254788 |
|  | 7510703 |
|  | 7512571 |
|  | 7513258 |
|  | 7518429 |
|  | 7535778 |
|  | 7536744 |
|  | 7589147 |
|  | 7629168 |
|  | 7657612 |
|  | 7681365 |
|  | 7682059 |
|  | 7761838 |
|  | 7799925 |
|  | 7999363 |
|  | 8084603 |
|  | 8155326 |
|  | 8290562 |
|  | 8384556 |
|  | 8386518 |
|  | 8443409 |
|  | 8596638 |
|  | 8627166 |
|  | 8628282 |
|  | 8657103 |
|  | 8660853 |
|  | 8662733 |
|  | 8885868 |
|  | 8892607 |
|  | 8905292 |
|  | 8910519 |
|  | 8918464 |
|  | 8995379 |
|  | 9164868 |
|  | 9174053 |
|  | 9178760 |
|  | 9207933 |
|  | 9231720 |
|  | 9278385 |
|  | 9281317 |
|  | 9299537 |
|  | 9314836 |
|  | 9344857 |
|  | 9398617 |
|  | 9489702 |
|  | 9516477 |
|  | 9535722 |
|  | 9600074 |
|  | 9697839 |
|  | 9712732 |
|  | 9729044 |
|  | 9730685 |
|  | 9743338 |
|  | 9891995 |
|  | 15532540 |
|  | 15879121 |
|  | 1689311 |
|  | 1708307 |
|  | 17116690 |
|  | 2831461 |
|  | 8181064 |
|  | 11997344 |
|  | 12782653 |
|  | 15256591 |
|  | 15998812 |
|  | 16565716 |
|  | 17309817 |
|  | 10488157 |
|  | 11780052 |
|  | 15270728 |
|  | 15592455 |
|  | 15702972 |
|  | 17081983 |
|  | 17148460 |
|  | 17242406 |
|  | 17372230 |
|  | 17419608 |
|  | 17561374 |
|  | 17562871 |
|  | 9045685 |
|  | 10646608 |
|  | 12471105 |
|  | 15308098 |
|  | 15489334 |
|  | 16380231 |
|  | 17531443 |
|  | 16973916 |
|  | 17252537 |
|  | 17432954 |
|  | 17524370 |
|  | 8106527 |
|  | 8183574 |
|  | 8392838 |
|  | 8402898 |
|  | 8910399 |

---

|  |  |
| --- | --- |
| Organism | Homo sapiens {Organism urn:agi-taxid:9606} |
|  | Mus musculus {Organism urn:agi-taxid:10090} |
|  | Rattus norvegicus {Organism urn:agi-taxid:10116} |
|  | Homo sapiens |
|  | Mus musculus |
|  | Rattus norvegicus |
|  | Bos taurus |
|  | Danio rerio |

---

|  |  |
| --- | --- |
| Mouse chromosome position | 2 80.97 cM |
|  | 2 92.0 cM |

---

|  |  |
| --- | --- |
| OMIM ID | 172420 |

---

|  |  |
| --- | --- |
| Rat chromosome position | 3q42 |

---

|  |  |
| --- | --- |
| Hugo ID | 9065 |
|  | HGNC:9065 |

---

|  |  |
| --- | --- |
| Human chromosome position | 20q12 |
|  | 20q12-q13.1 |

---

|  |  |
| --- | --- |
| Swiss-Prot Accession | P19174 |
|  | Q4LE43 |
|  | Q9UFY1 |
|  | P19174.1 |
|  | Q62077 |
|  | Q62077.2 |
|  | P10686 |
|  | P10686.1 |
|  | B7ZLY7 |
|  | B9EGH4 |
|  | E1P5W4 |
|  | Q2V575 |
|  | Q6P1G1 |
|  | A2A284 |
|  | A2A4A7 |
|  | Q6LC80 |
|  | Q2M4I9 |
|  | Q3TE89 |
|  | Q3TUJ5 |
|  | Q3UZ68 |
|  | Q792T1 |
|  | Q9Z2S5 |
|  | PLCG1\_HUMAN |
|  | PLCG1\_MOUSE |
|  | PLCG1\_RAT |
|  | A2A284\_HUMAN |
|  | Q4LE43\_HUMAN |
|  | A2A4A7\_MOUSE |
|  | Q9BQY5 |

---

|  |  |
| --- | --- |
| PIR ID | A36466 |
|  | A31317 |
|  | T14776 |

---

|  |  |
| --- | --- |
| GenBank ID | NC\_000020 |
|  | XR\_001754293 |
|  | XR\_001754294 |
|  | XR\_001754295 |
|  | XR\_001754296 |
|  | XM\_005260438 |
|  | XP\_005260495 |
|  | XM\_011528867 |
|  | XP\_011527169 |
|  | NM\_002660 |
|  | NP\_002651 |
|  | NM\_182811 |
|  | NP\_877963 |
|  | XM\_011528865 |
|  | XP\_011527167 |
|  | AL022394 |
|  | CH471077 |
|  | EAW75989 |
|  | EAW75990 |
|  | EAW75991 |
|  | EAW75992 |
|  | EAW75993 |
|  | DQ297143 |
|  | ABB84466 |
|  | U80983 |
|  | AAB70663 |
|  | AB210028 |
|  | BAE06110 |
|  | AF147391 |
|  | AK297370 |
|  | BAG59815 |
|  | AK309606 |
|  | AL110247 |
|  | CAB53696 |
|  | BC136466 |
|  | AAI36467 |
|  | BC144136 |
|  | AAI44137 |
|  | BX415739 |
|  | BX434193 |
|  | M34667 |
|  | AAA36452 |
|  | P19174 |
|  | NC\_000068 |
|  | NM\_021280 |
|  | NP\_067255 |
|  | AH007341 |
|  | AAD13798 |
|  | AL590389 |
|  | AY902326 |
|  | AAX90611 |
|  | CH466551 |
|  | EDL06290 |
|  | EDL06291 |
|  | EDL06292 |
|  | HB877232 |
|  | CBF62965 |
|  | HB895866 |
|  | CBF74731 |
|  | HC934641 |
|  | CBU87841 |
|  | HC953275 |
|  | CBU96788 |
|  | AF027185 |
|  | AAD09344 |
|  | AK134041 |
|  | BAE21990 |
|  | AK160731 |
|  | BAE35976 |
|  | AK162809 |
|  | AK169695 |
|  | BAE41310 |
|  | AK169777 |
|  | BAE41359 |
|  | AK185701 |
|  | BC057161 |
|  | BC065091 |
|  | AAH65091 |
|  | X95346 |
|  | CAA64639 |
|  | Q62077 |
|  | NC\_005102 |
|  | NM\_013187 |
|  | NP\_037319 |
|  | AC\_000071 |
|  | AAHX01026215 |
|  | AC128986 |
|  | CH474005 |
|  | EDL96614 |
|  | EDL96615 |
|  | J03806 |
|  | AAA41921 |
|  | P10686 |
|  | XR\_936550 |
|  | XM\_011528866 |
|  | XP\_011527168 |
|  | NC\_018931 |
|  | AMYH02037082 |
|  | AMYH02037083 |
|  | AC\_000024 |
|  | AAHY01012440 |
|  | XR\_244143 |
|  | AC\_000152 |
|  | ABBA01052574 |
|  | AABR06027396 |
|  | CAA18537 |
|  | CAM28260 |
|  | CAM23067 |
|  | CAM23068 |
|  | CAM23069 |
|  | NT\_011362 |
|  | NW\_001838666 |
|  | NW\_927339 |
|  | AC\_000063 |
|  | Q4LE43 |
|  | Q6LC80 |
|  | Q9UFY1 |
|  | NT\_039207 |
|  | NW\_001030712 |
|  | AF027186 |
|  | AF027187 |
|  | Q2M4I9 |
|  | Q3TE89 |
|  | Q3TUJ5 |
|  | Q3UZ68 |
|  | Q792T1 |
|  | Q9Z2S5 |
|  | NW\_047659 |
|  | NW\_001084817 |
|  | Q9BQY5 |
|  | BM121087 |
|  | CAC36283 |
|  | NW\_000179 |
|  | 130226 |
|  | 206324 |
|  | 4103747 |
|  | 4103753 |
|  | 123782930 |
|  | 123788798 |
|  | 123793748 |
|  | 123796465 |
|  | 1246803 |
|  | 40675439 |
|  | 62635484 |
|  | 74137194 |
|  | 74141388 |
|  | 74146600 |
|  | 74146760 |
|  | 81870156 |
|  | 81894283 |
|  | 85700388 |
|  | AI894140 |
|  | 119596396 |
|  | 119596397 |
|  | 119596398 |
|  | 121948502 |
|  | 130225 |
|  | 190038 |
|  | 2408215 |
|  | 3336979 |
|  | 5817197 |
|  | 68533111 |
|  | 74709702 |
|  | 74752259 |
|  | 74753302 |
|  | 82583660 |
|  | NT\_086910 |
|  | 130224 |
|  | 639 |
|  | CAA68406 |
|  | NC\_007311 |
|  | NM\_174425 |
|  | NP\_776850 |
|  | NW\_001493162 |
|  | P08487 |
|  | Y00301 |
|  | AW279908 |
|  | AW280848 |
|  | AW281486 |
|  | AW281801 |
|  | 27883903 |
|  | 27883905 |
|  | 27883907 |
|  | 82098722 |
|  | 82098725 |
|  | 82098727 |
|  | AAO23907 |
|  | AAO23908 |
|  | AAO23909 |
|  | AY163168 |
|  | AY163169 |
|  | AY163170 |
|  | NC\_007134 |
|  | NM\_194407 |
|  | NP\_919388 |
|  | NW\_001512488 |
|  | Q804J4 |
|  | Q804J5 |
|  | Q804J6 |
|  | 119596395 |
|  | 119596399 |
|  | 123236076 |
|  | 148674343 |
|  | 148674344 |
|  | 148674345 |
|  | 149043040 |
|  | 149043041 |

---

|  |  |
| --- | --- |
| Swiss-Prot ID | PLCG1\_HUMAN |
|  | PLCG1\_MOUSE |
|  | PLCG1\_RAT |
|  | Q9UFY1\_HUMAN |
|  | A2A284\_HUMAN |
|  | A2A4A7\_MOUSE |
|  | Q4LE43\_HUMAN |

---

|  |  |
| --- | --- |
| Cell Localization | Cell projection |
|  | Lamellipodium |
|  | Ruffle |
|  | Cell projection, lamellipodium |
|  | Cell projection, ruffle |
|  | Plasma membrane |
|  | Cytoplasm |

---

|  |  |
| --- | --- |
| Ensembl ID | ENSG00000124181 |
|  | ENSP00000244007.3 |
|  | ENST00000244007.7 |
|  | ENSP00000362368.1 |
|  | ENST00000373271.5 |
|  | ENSMUSG00000016933 |
|  | ENSMUSP00000099404.1 |
|  | ENSMUST00000103115.7 |
|  | ENSRNOG00000051490 |
|  | ENSRNOP00000069822.1 |
|  | ENSRNOT00000078909.1 |
|  | ENSP00000244007 |
|  | ENST00000244007 |
|  | ENSP00000362368 |
|  | ENST00000373271 |
|  | ENSMUSP00000099404 |
|  | ENSMUST00000103115 |
|  | ENSRNOP00000069822 |
|  | ENSRNOT00000078909 |
|  | ENSRNOG00000016340 |
|  | ENSRNOP00000022276 |
|  | ENSRNOT00000022276 |
|  | ENSP00000362369 |
|  | ENST00000373272 |

---

|  |  |
| --- | --- |
| MGI ID | MGI:97615 |
|  | 97615 |
|  | 1329022 |
|  | 2139113 |

---

|  |  |
| --- | --- |
| RGD ID | 3347 |

---

|  |  |
| --- | --- |
| Unigene ID | Hs.268177 |
|  | Mm.44463 |
|  | Rn.11243 |
|  | Bt.307 |
|  | Dr.120304 |

---

|  |  |
| --- | --- |
| Homologene ID | 1997 |

---

|  |  |
| --- | --- |
| IPI ID | IPI00192532 |
|  | IPI00016736 |
|  | IPI00383849 |
|  | IPI00121089 |
|  | IPI00745182 |
|  | IPI00653317 |
|  | IPI00654932 |
|  | IPI00757004 |
|  | IPI00753388 |
|  | IPI00212420 |

---

|  |  |
| --- | --- |
| Microarray ID | 98290\_at |

---

|  |  |
| --- | --- |
| Homo sapiens Chromosome position | 20q12-q13.1 |

---

|  |  |
| --- | --- |
| Ensembl Transcript ID | ENST00000373272 |
|  | ENST00000244007 |
|  | ENST00000373271 |

---

|  |  |
| --- | --- |
| ENSEMBL | ENSG00000124181 |

---

|  |  |
| --- | --- |
| FunctionalClass | insulin receptor binding |
|  | phosphoinositide phospholipase C |
|  | phospholipase C |
|  | protein binding |
|  | receptor signaling protein |
|  | calcium ion binding |
|  | hydrolase |
|  | signal transducer |
|  | phosphoprotein binding |

---

|  |  |
| --- | --- |
| Bos taurus Chromosome position | 13 |

---

|  |  |
| --- | --- |
| Mus musculus Chromosome position | 2 93.0 cM |
|  | 2 92.0 cM |
|  | 2 H2 |

---

|  |  |
| --- | --- |
| KEGG pathway | Calcium signaling pathway |
|  | ErbB signaling pathway |
|  | Fc epsilon RI signaling pathway |
|  | Glioma |
|  | Inositol phosphate metabolism |
|  | Leukocyte transendothelial migration |
|  | Natural killer cell mediated cytotoxicity |
|  | Non-small cell lung cancer |
|  | Phosphatidylinositol signaling system |
|  | VEGF signaling pathway |

---

|  |  |
| --- | --- |
| Rattus norvegicus Chromosome position | 3q42 |

---

|  |  |
| --- | --- |
| Ensembl Gene ID | ENSG00000124181 |

---

|  |  |
| --- | --- |
| ProteinAtlas NormalTissue | Tissue: adrenal gland; Cell type: glandular cells; Level: Strong; Expression type: Staining; Reliability: Uncertain |
|  | Tissue: appendix; Cell type: glandular cells; Level: Strong; Expression type: Staining; Reliability: Uncertain |
|  | Tissue: appendix; Cell type: lymphoid tissue; Level: Strong; Expression type: Staining; Reliability: Uncertain |
|  | Tissue: bone marrow; Cell type: hematopoietic cells; Level: Strong; Expression type: Staining; Reliability: Uncertain |
|  | Tissue: breast; Cell type: glandular cells; Level: Moderate; Expression type: Staining; Reliability: Uncertain |
|  | Tissue: bronchus; Cell type: respiratory epithelial cells; Level: Moderate; Expression type: Staining; Reliability: Uncertain |
|  | Tissue: cerebellum; Cell type: cells in granular layer; Level: Strong; Expression type: Staining; Reliability: Uncertain |
|  | Tissue: cerebellum; Cell type: cells in molecular layer; Level: Strong; Expression type: Staining; Reliability: Uncertain |
|  | Tissue: cerebellum; Cell type: Purkinje cells; Level: Strong; Expression type: Staining; Reliability: Uncertain |
|  | Tissue: cerebral cortex; Cell type: glial cells; Level: Strong; Expression type: Staining; Reliability: Uncertain |
|  | Tissue: cerebral cortex; Cell type: neuronal cells; Level: Strong; Expression type: Staining; Reliability: Uncertain |
|  | Tissue: cervix, uterine; Cell type: glandular cells; Level: Moderate; Expression type: Staining; Reliability: Uncertain |
|  | Tissue: cervix, uterine; Cell type: squamous epithelial cells; Level: Strong; Expression type: Staining; Reliability: Uncertain |
|  | Tissue: colon; Cell type: glandular cells; Level: Strong; Expression type: Staining; Reliability: Uncertain |
|  | Tissue: duodenum; Cell type: glandular cells; Level: Strong; Expression type: Staining; Reliability: Uncertain |
|  | Tissue: epididymis; Cell type: glandular cells; Level: Strong; Expression type: Staining; Reliability: Uncertain |
|  | Tissue: esophagus; Cell type: squamous epithelial cells; Level: Strong; Expression type: Staining; Reliability: Uncertain |
|  | Tissue: fallopian tube; Cell type: glandular cells; Level: Moderate; Expression type: Staining; Reliability: Uncertain |
|  | Tissue: gall bladder; Cell type: glandular cells; Level: Strong; Expression type: Staining; Reliability: Uncertain |
|  | Tissue: heart muscle; Cell type: myocytes; Level: Strong; Expression type: Staining; Reliability: Uncertain |
|  | Tissue: hippocampus; Cell type: glial cells; Level: Strong; Expression type: Staining; Reliability: Uncertain |
|  | Tissue: hippocampus; Cell type: neuronal cells; Level: Strong; Expression type: Staining; Reliability: Uncertain |
|  | Tissue: kidney; Cell type: cells in glomeruli; Level: Strong; Expression type: Staining; Reliability: Uncertain |
|  | Tissue: kidney; Cell type: cells in tubules; Level: Strong; Expression type: Staining; Reliability: Uncertain |
|  | Tissue: lateral ventricle; Cell type: glial cells; Level: Strong; Expression type: Staining; Reliability: Uncertain |
|  | Tissue: lateral ventricle; Cell type: neuronal cells; Level: Strong; Expression type: Staining; Reliability: Uncertain |
|  | Tissue: liver; Cell type: bile duct cells; Level: Moderate; Expression type: Staining; Reliability: Uncertain |
|  | Tissue: liver; Cell type: hepatocytes; Level: Strong; Expression type: Staining; Reliability: Uncertain |
|  | Tissue: lung; Cell type: macrophages; Level: Strong; Expression type: Staining; Reliability: Uncertain |
|  | Tissue: lung; Cell type: pneumocytes; Level: Moderate; Expression type: Staining; Reliability: Uncertain |
|  | Tissue: lymph node; Cell type: germinal center cells; Level: Moderate; Expression type: Staining; Reliability: Uncertain |
|  | Tissue: lymph node; Cell type: non-germinal center cells; Level: Strong; Expression type: Staining; Reliability: Uncertain |
|  | Tissue: nasopharynx; Cell type: respiratory epithelial cells; Level: Strong; Expression type: Staining; Reliability: Uncertain |
|  | Tissue: oral mucosa; Cell type: squamous epithelial cells; Level: Strong; Expression type: Staining; Reliability: Uncertain |
|  | Tissue: ovary; Cell type: follicle cells; Level: Strong; Expression type: Staining; Reliability: Uncertain |
|  | Tissue: ovary; Cell type: ovarian stroma cells; Level: Strong; Expression type: Staining; Reliability: Uncertain |
|  | Tissue: pancreas; Cell type: exocrine glandular cells; Level: Moderate; Expression type: Staining; Reliability: Uncertain |
|  | Tissue: pancreas; Cell type: islets of Langerhans; Level: Moderate; Expression type: Staining; Reliability: Uncertain |
|  | Tissue: parathyroid gland; Cell type: glandular cells; Level: Strong; Expression type: Staining; Reliability: Uncertain |
|  | Tissue: placenta; Cell type: decidual cells; Level: Moderate; Expression type: Staining; Reliability: Uncertain |
|  | Tissue: placenta; Cell type: trophoblastic cells; Level: Strong; Expression type: Staining; Reliability: Uncertain |
|  | Tissue: prostate; Cell type: glandular cells; Level: Moderate; Expression type: Staining; Reliability: Uncertain |
|  | Tissue: rectum; Cell type: glandular cells; Level: Strong; Expression type: Staining; Reliability: Uncertain |
|  | Tissue: salivary gland; Cell type: glandular cells; Level: Moderate; Expression type: Staining; Reliability: Uncertain |
|  | Tissue: seminal vesicle; Cell type: glandular cells; Level: Strong; Expression type: Staining; Reliability: Uncertain |
|  | Tissue: skeletal muscle; Cell type: myocytes; Level: Strong; Expression type: Staining; Reliability: Uncertain |
|  | Tissue: skin; Cell type: epidermal cells; Level: Moderate; Expression type: Staining; Reliability: Uncertain |
|  | Tissue: small intestine; Cell type: glandular cells; Level: Strong; Expression type: Staining; Reliability: Uncertain |
|  | Tissue: smooth muscle; Cell type: smooth muscle cells; Level: Strong; Expression type: Staining; Reliability: Uncertain |
|  | Tissue: spleen; Cell type: cells in red pulp; Level: Strong; Expression type: Staining; Reliability: Uncertain |
|  | Tissue: spleen; Cell type: cells in white pulp; Level: Strong; Expression type: Staining; Reliability: Uncertain |
|  | Tissue: stomach, lower; Cell type: glandular cells; Level: Strong; Expression type: Staining; Reliability: Uncertain |
|  | Tissue: stomach, upper; Cell type: glandular cells; Level: Strong; Expression type: Staining; Reliability: Uncertain |
|  | Tissue: testis; Cell type: cells in seminiferus ducts; Level: Strong; Expression type: Staining; Reliability: Uncertain |
|  | Tissue: testis; Cell type: Leydig cells; Level: Moderate; Expression type: Staining; Reliability: Uncertain |
|  | Tissue: thyroid gland; Cell type: glandular cells; Level: Moderate; Expression type: Staining; Reliability: Uncertain |
|  | Tissue: tonsil; Cell type: germinal center cells; Level: Strong; Expression type: Staining; Reliability: Uncertain |
|  | Tissue: tonsil; Cell type: non-germinal center cells; Level: Strong; Expression type: Staining; Reliability: Uncertain |
|  | Tissue: tonsil; Cell type: squamous epithelial cells; Level: Strong; Expression type: Staining; Reliability: Uncertain |
|  | Tissue: urinary bladder; Cell type: urothelial cells; Level: Strong; Expression type: Staining; Reliability: Uncertain |
|  | Tissue: uterus, post-menopause; Cell type: cells in endometrial stroma; Level: Strong; Expression type: Staining; Reliability: Uncertain |
|  | Tissue: uterus, post-menopause; Cell type: glandular cells; Level: Moderate; Expression type: Staining; Reliability: Uncertain |
|  | Tissue: uterus, pre-menopause; Cell type: cells in endometrial stroma; Level: Moderate; Expression type: Staining; Reliability: Uncertain |
|  | Tissue: uterus, pre-menopause; Cell type: glandular cells; Level: Strong; Expression type: Staining; Reliability: Uncertain |
|  | Tissue: vagina; Cell type: squamous epithelial cells; Level: Moderate; Expression type: Staining; Reliability: Uncertain |
|  | Tissue: vulva/anal skin; Cell type: epidermal cells; Level: Strong; Expression type: Staining; Reliability: Uncertain |
|  | Tissue: cervix |
|  | uterine; Cell type: squamous epithelial cells; Level: Strong; Expression type: Staining; Reliability: Uncertain |

---

|  |  |
| --- | --- |
| Danio rerio Chromosome position | LG 23 |

---

|  |  |
| --- | --- |
| EnzymeConsortium | 3.1.4.11 |

---
